# Identification of novel genetic loci for risk of multiple myeloma by functional annotation

**DOI:** 10.1038/s41375-023-02022-8

**Published:** 2023-09-18

**Authors:** Angelica Macauda, Klara Briem, Alyssa Clay-Gilmour, Wendy Cozen, Asta Försti, Matteo Giaccherini, Chiara Corradi, Juan Sainz, Yasmeen Niazi, Rob ter Horst, Yang Li, Mihai G. Netea, Ulla Vogel, Kari Hemminki, Susan L. Slager, Judit Varkonyi, Vibeke Andersen, Elzbieta Iskierka-Jazdzewska, Joaquin Mártinez-Lopez, Jan Zaucha, Nicola J. Camp, S. Vincent Rajkumar, Agnieszka Druzd-Sitek, Parveen Bhatti, Stephen J. Chanock, Shaji K. Kumar, Edyta Subocz, Grzegorz Mazur, Stefano Landi, Mitchell J. Machiela, Andrés Jerez, Aaron D. Norman, Michelle A. T. Hildebrandt, Katalin Kadar, Sonja I. Berndt, Elad Ziv, Gabriele Buda, Arnon Nagler, Charles Dumontet, Malgorzata Raźny, Marzena Watek, Aleksandra Butrym, Norbert Grzasko, Marek Dudzinski, Malwina Rybicka-Ramos, Eva-Laure Matera, Ramón García-Sanz, Hartmut Goldschmidt, Krzysztof Jamroziak, Artur Jurczyszyn, Esther Clavero, Graham G. Giles, Matteo Pelosini, Daria Zawirska, Marcin Kruszewski, Herlander Marques, Eva Haastrup, José Manuel Sánchez-Maldonado, Uta Bertsch, Marcin Rymko, Marc-Steffen Raab, Elizabeth E. Brown, Jonathan N. Hofmann, Celine Vachon, Daniele Campa, Federico Canzian

**Affiliations:** 1 Genomic Epidemiology Group, German Cancer Research Center (DKFZ), Heidelberg, Germany; 2 Department of Epidemiology & Biostatistics, Arnold School of Public Health, University of South Carolina, Columbia, SC USA; 3 Division of Hematology/Oncology, Division of Hematology/Oncology, Department of Medicine, School of Medicine, Department of Pathology, School of Medicine, Susan and Henry Samueli College of Health Sciences, Chao Family Comprehensive Cancer Center, University of California at Irvine, Irvine, CA USA; 4 Division of Pediatric Neurooncology, German Cancer Research Center (DKFZ), Heidelberg, Germany; 5 Hopp Children’s Cancer Center (KiTZ), Heidelberg, Germany Division of Pediatric Neurooncology, German Cancer Research Center (DKFZ), German Cancer Consortium (DKTK), Heidelberg, Germany; 6 Department of Biology, University of Pisa, Pisa, Italy; 7 Genomic Oncology Area, GENYO. Centre for Genomics and Oncological Research: Pfizer / University of Granada / Andalusian Regional Government, PTS, Granada, Spain; 8 Instituto de Investigación Biosanitaria IBs.Granada, Granada, Spain; 9 Department of Biochemistry and Molecular Biology I, University of Granada, Granada, Spain; 10 Department of Internal Medicine and Radboud Center for Infectious Diseases, Radboud University Medical Center, Nijmegen, The Netherlands; 11 CeMM Research Center for Molecular Medicine of the Austrian Academy of Sciences, Vienna, Austria; 12 Centre for Individualised Infection Medicine (CiiM) & TWINCORE, joint ventures between the Helmholtz-Centre for Infection Research (HZI) and the Hannover Medical School (MHH), Hannover, Germany; 13 Department for Immunology & Metabolism, Life and Medical Sciences Institute (LIMES), University of Bonn, Bonn, Germany; 14 National Research Centre for the Working Environment, Copenhagen, Denmark; 15 Biomedical Center, Faculty of Medicine, Charles University Pilsen, Pilsen, Czech Republic; 16 Division of Cancer Epidemiology, German Cancer Research Center (DKFZ), Heidelberg, Germany; 17 Division of Biomedical Statistics & Informatics, Department of Health Sciences Research, Mayo Clinic, Rochester, MN USA; 18 Department of Hematology, Semmelweis University, Budapest, Hungary; 19 Molecular Diagnostics and Clinical Research Unit, Institute of Regional Health Research, University Hospital of Southern Denmark, Odense, Denmark; 20 Institute of Regional Research, University of Southern Denmark, Odense, Denmark; 21 Institute of Molecular Medicine, University of Southern Denmark, Odense, Denmark; 22 Department of Hematology, Transplantology and Internal Medicine, Medical University of Warsaw, Warsaw, Poland; 23 Hospital Universitario 12 de Octubre, Instituto de Investigación del Hospital Universitario 12 de Octubre, 28041 Madrid, Spain; 24 Department of Haematology & Transplantology, Medical University of Gdańsk, Gdańsk, Poland; 25 Division of Hematology and Huntsman Cancer Institute, University of Utah, Salt Lake City, UT USA; 26 Division of Hematology, Department of Internal Medicine, Mayo Clinic, Rochester, MN USA; 27 Department of Lymphoproliferative Diseases, Maria Skłodowska-Curie National Research Institute of Oncology, Warsaw, Poland; 28 Cancer Control Research, BC Cancer, Vancouver, BC Canada; 29 Program in Epidemiology, Public Health Sciences Division, Fred Hutchinson Cancer Research Center, Seattle, WA USA; 30 Division of Cancer Epidemiology and Genetics, National Cancer Institute, National Institutes of Health, Bethesda, MD USA; 31 Department of Hematology, Warmian-Masurian Cancer Center of The Ministry Of The Interior And Administration’s Hospital, Olsztyn, Poland; 32 Wroclaw Medical University, Wroclaw, Poland; 33 Hematology and Medical Oncology Department, University Hospital Morales Meseguer, IMIB, Murcia, Spain; 34 Division of Epidemiology, Department of Health Sciences Research, Mayo Clinic, Rochester, MN USA; 35 Department of Lymphoma - Myeloma, Division of Cancer Medicine, The University of Texas MD Anderson Cancer Center, Houston, TX USA; 36 St. John’s Hospital, Budapest, Hungary; 37 Department of Medicine, University of California San Francisco Helen Diller Family Comprehensive Cancer Center, San Francisco, CA USA; 38 Hematology Unit, Department of Clinical and Experimental Medicine, University of Pisa, Pisa, Italy; 39 Hematology Division Chaim Sheba Medical Center, Tel Hashomer, Israel; 40 INSERM 1052/CNRS 5286, CRCL, Lyon, France; 41 Department of Hematology, Rydygier Specialistic Hospital, Cracow, Poland; 42 Department of Hematology, Institute of Hematology and Transfusion Medicine, Warsaw, Poland; 43 Department of Hematology, Holycross Cancer Center, Kielce, Poland; 44 Alfred Sokolowski Specialist Hospital in Walbrzych Oncology Support Centre for Clinical Trials, Wałbrzych, Poland; 45 Department of Experimental Hematooncology, Medical University of Lublin, Lublin, Poland; 46 Institute of Medical Sciences, College of Medical Sciences, University of Rzeszow, Rzeszow, Poland; 47 Department of Hematology, Specialist Hospital No.1 in Bytom, Academy of Silesia, Faculty of Medicine, Katowice, Poland; 48 University Hospital of Salamanca, Diagnostic Laboratory Unit in Hematology, University Hospital of Salamanca, IBSAL, CIBERONC, Centro de Investigación del Cáncer-IBMCC (USAL-CSIC), Salamanca, Spain; 49 Department of Internal Medicine V, Heidelberg University Hospital, Heidelberg, Germany; 50 National Centre for Tumour Diseases (NCT), University Hospital Heidelberg, Heidelberg, Germany; 51 National Centre for Tumour Diseases (NCT), University Hospital Heidelberg, Heidelberg, Germany. GMMG Study Group at University Hospital Heidelberg, Heidelberg, Germany; 52 Hematology Department, Jagiellonian University Medical College, Cracow, Poland; 53 Hematology Department, Virgen de las Nieves University Hospital, Granada, Spain; 54 Cancer Epidemiology Division, Cancer Council Victoria, Melbourne, VIC Australia; 55 Centre for Epidemiology and Biostatistics, School of Population and Global Health, The University of Melbourne, Melbourne, VIC Australia; 56 Precision Medicine, School of Clinical Sciences at Monash Health, Monash University, Clayton, VIC Australia; 57 U.O. Dipartimento di Ematologia, Azienda USL Toscana Nord Ovest, Livorno, Italy; 58 Department of Hematology, University Hospital, Crakow, Poland; 59 Department of Hematology, University Hospital No. 2 in Bydgoszcz, Bydgoszcz, Poland; 60 Life and Health Sciences Research Institute (ICVS), School of Health Sciences, University of Minho, Braga, Portugal; 61 ICVS/3B’s - PT Government Associate Laboratory, Braga/Guimarães, Portugal; 62 Department of Clinical Immunology, the Bloodbank, Rigshospitalet, Copenhagen University Hospital, København, Denmark; 63 Department of Hematology, Provincial Polyclinical Hospital in Torun, Torun, Poland; 64 Department of Pathology, School of Medicine at the University of Alabama, Birmingham, AL USA

**Keywords:** Risk factors, Genetics research

## To the Editor:

Multiple myeloma (MM) is one of the most common hematological malignancies, accounting for 20% of all newly diagnosed hematological cancers [1]. The most recent data from Cancer Today show that in 2020 the number of new MM cases was 176,404 worldwide (https://gco.iarc.fr/today/home).

Established risk factors for MM include age, male sex, African ancestry, obesity, chronic inflammation, exposure to pesticides, organic solvents, and radiation [2]. Familial aggregation of MM and its precursor monoclonal gammopathy of undetermined significance (MGUS) suggests that genetic factors play a role in risk of MM as well [3]. Genetic variability has been identified as a risk factor for MM, including 25 common genetic loci identified in genome-wide association studies (GWAS). However, estimates of heritability show that many more loci remain to be found [4].

A key question is therefore how to find new causative variants. The stringent significance threshold usually used in GWAS (*p* < 5 × 10^−8^) accounts for the many statistical tests being performed but may result in false negatives. Reducing the number of tests will relax the required significance threshold, thereby increasing statistical power to detect associations with MM risk for each SNP. One strategy for reducing the number of tests is to examine SNPs with higher prior probability of association according to meaningful biological criteria. We looked for novel MM risk loci using a two-phase large-scale association study, prioritizing polymorphisms with predicted functional impact, a strategy that has been used for other cancers and led to the discovery of new loci [5–7] It is well known that functional variants are indeed more likely to be associated with disease development [8].

We used data from the International Lymphoma Epidemiology Consortium (InterLymph) for discovery and from the German-speaking Myeloma Multicenter Group (GMMG), the International Multiple Myeloma rESEarch (IMMEnSE) consortium, as well as summary statistics from the FinnGen study for replication, for a total of 5982 MM cases and 266,173 controls. Detailed characteristics of the study populations are shown in the supplementary methods and supplementary table 1.

Candidate SNPs to be replicated were selected based on their association with MM risk and their functional role. First, we obtained summary results including odds ratios (OR), 95% confidence intervals (95%-CI), and *p*-values of the top SNPs of the InterLymph GWAS. Subsequently, all SNPs in the MM data set from InterLymph with *p* < 5 × 10^−4^ (*N* = 4396) were looked up in the first replication dataset, the GMMG GWAS. We did not consider SNPs from 15 loci that were reported to be associated at genome-wide significance level in previous GWASs. All SNPs with significant *p*-values (*p* < 0.05) in the GMMG GWAS and ORs going in the same direction in both datasets were selected. The next step was annotating the selected SNPs (*N* = 136) for their predicted function, using several suitable bioinformatic tools and databases. Specifically, we looked at expression and splicing quantitative trait loci (eQTLs and sQTLs), SNPs located in transcription factor binding sites (TFBS), long non-coding RNA (lncSNPs), SNPs that are located within enhancers, and polymorphisms located in gene coding regions (missense, stop-gain, stop-loss, synonymous SNPs). Supplementary Table 2 shows the details of the 136 SNPs and their predicted functional characterization. The resulting list from all annotations was pruned for linkage disequilibrium (LD) using the LDlink portal (https://ldlink.nci.nih.gov/). Only SNPs with r^2^ < 0.6 among them were kept, resulting in a total of 12 independent loci on 9 chromosomes. Replication in IMMEnSE and FinnGen was attempted for SNPs showing association with risk in the meta-analysis between InterLymph and GMMG GWAS and at least one in silico functional annotation. After exclusion of SNPs that had already been analysed in IMMEnSE in the context of previous projects and already shown not to be significantly associated with MM risk (on chromosomes 6, 8, 12 and 21), 4 SNPs showed to have low *p*-value of association with MM risk and had at least one functional prediction annotation (rs12038685, rs2664188, rs12652920, rs28199), which were therefore chosen for replication in IMMEnSE (Supplementary Table 3). An in-depth description of the SNP functional annotation and selection, as well as the technical details of the genotyping and quality control, can be found in the Supplementary Methods.

Analysis of association between each SNP and MM risk was carried out with logistic regression models, by estimating ORs, their 95%-CI, and associated *p*-value. The analyses were adjusted for age (at diagnosis for MM cases and recruitment for controls), sex, and the 10 first principal components for GWAS data, or country of origin in IMMEnSE, which lacks GWAS data. All meta-analyses were conducted with R, using a fixed-effect model between summary statistics of the different studies. The *I*² statistic was computed to quantify heterogeneity across studies.

**Fig. 1:**
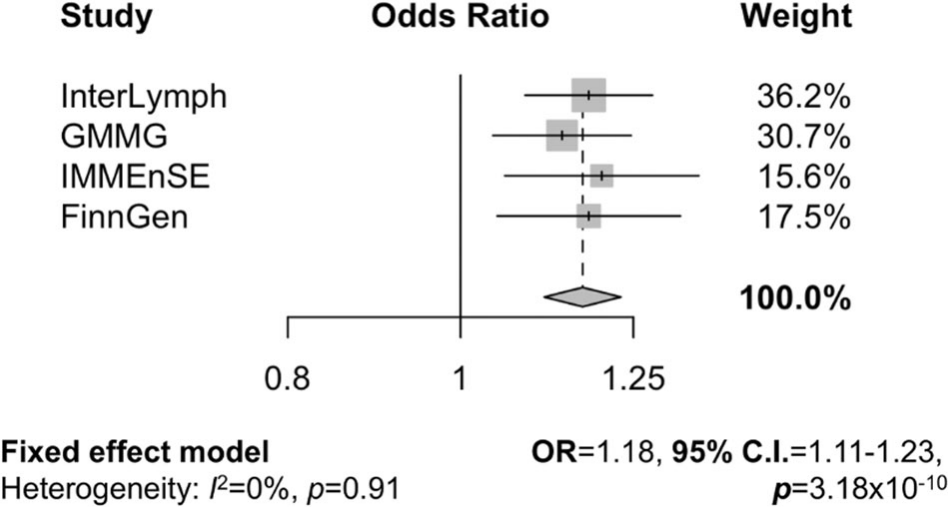
Meta-analysis result of rs28199. Forest plot of the meta-analysis using a fixed effects model across all four datasets. Heterogeneity was assessed using the *I*^2^ statistic. OR = odds ratio, 95% CI = 95% confidence interval.

rs28199, on chromosome 5, was associated with MM risk in IMMEnSE (OR = 1.19, 95% C.I. = 0.72–0.97, *p* = 0.018) and FinnGen (1.17, 95% C.I. = 1.05–1.31, *p* = 0.014). The G allele of this SNP resulted to be significantly associated with increased MM risk at a genome-wide level in the meta-analysis of the four datasets (OR = 1.18, 95% C.I. = 1.11–1.23, *p* = 3.18 × 10^−10^) with no heterogeneity among the studies (*I*^2^ = 0) (Table 1, Fig. 1).

**Table 1:** Association results of the SNPs selected for replication in IMMENSE.

SNP	Study	OR^a^	95% CI^b^	*p*-value	Functional annotation^c^
rs12038685	InterLymph	1.22	1.11–1.34	5.04 × 10^-5^	TBFS
	GMMG	1.11	1.00–1.23	0.046	
	FinnGen	1.2	1.07–1.32	0.004	
	IMMEnSE	0.94	0.81–1.09	0.409	
	Meta-analysis	1.18	1.10–1.25	2.07 × 10^7^	
rs2664188	InterLymph	1.18	1.09–1.28	8.14 × 10^−5^	eQTL
	GMMG	1.09	1.00–1.09	0.048	
	FinnGen	1.01	0.89–1.12	0.808	
	IMMEnSE	0.96	0.84–1.10	0.58	
	Meta-analysis	0.91	0.85–0.97	0.002	
rs12652920	InterLymph	0.83	0.75–0.91	1.62 × 10^−4^	TBFS
	GMMG	0.90	0.81–0.99	0.031	
	FinnGen	1.09	0.96–1.22	0.174	
	IMMEnSE	0.99	0.84–1.15	0.875	
	Meta-analysis	0.91	0.86–0.97	0.0001	
rs28199	InterLymph	1.18	1.10–1.28	7.31 × 10^−5^	TBFS
	GMMG	1.14	1.04–1.25	0.004	
	FinnGen	1.18	1.05–1.31	0.014	
	IMMEnSE	1.20	1.03–1.39	0.018	
	Meta-analysis	1.18	1.11–1.23	3.18 × 10^−10^	

^a^*OR* odds ratio.

^b^*95%C.I.* 95% confidence interval.

^c^TFBS: the SNP is predicted to alter one or more transcription factor binding site(s); eQTL: the SNP is predicted to be a quantitative trait locus in whole blood or EBV-transformed B-lymphocyte cell lines.

This polymorphism was selected for being predicted to affect the binding site of three transcription factors: IRF1, STAT2_STAT1 and FOXP1. The strongest effect of the SNP was calculated for IRF1 (interferon regulatory factor 1), a protein member of the IRF family which was first recognized for its role as activator of genes involved in both innate and acquired immune responses. IRF-1 activates a set of target genes associated with regulation of cell cycle, apoptosis and the immune response [9, 10]. According to the SNP2TFBS database, rs28199 is predicted to modify a binding site of IRF1 leading to a stronger bond, which could in turn result in oncogenesis considering the set of genes that IRF1 regulates. The minor allele of rs28199 is located within a regulatory region which according to the variant effect predictor tool (https://www.ensembl.org/info/docs/tools/vep/index.html) and HaploReg (https://pubs.broadinstitute.org/mammals/haploreg/haploreg.php) binds the CTCF protein, a highly conserved zinc finger with various cellular regulatory role. CTCF binding perturbations cause different types of 3D genome reorganization and may cause the activation of the neighboring oncogenes [11]. Among the genes that CTCF regulates there is *STK10*, which encodes for a serine/threonine-protein kinase, highly expressed in hematopoietic tissue [12]. In various lymphoid cells rs28199-G is associated with an increased expression of *STK10*. Overexpression of *STK10* has been reported in several cancer types, including acute myeloid leukemia (AML), another blood malignancy [13, 14].

We used data from the 500 Functional Genomics cohort from the Human Functional Genomics Project (HFGP; http://www.humanfunctionalgenomics.org/site/) to explore the possible role in modulating immune response of the four SNPs selected for the final replication steps. Namely, we tested if any of the SNPs of interest were cytokine expression quantitative trait loci (cQTL) using data from in vitro stimulation experiments, as well as absolute numbers of 91 blood-derived cell populations and 103 serum or plasmatic inflammatory proteins. The cQTL analyses showed that rs28199-G is also associated with an increased blood level of Interleukin-6 (IL-6) (beta=0.075, *p* = 0.002). IL-6 is a cytokine with a well established role as a growth and survival factor in MM [15]. Specifically, in line with our results, an increased level of IL-6 contributes to the pleiotropic effects of IL-6 regarding proliferation, survival, drug resistance, and migration of MM cells, thereby facilitating disease progression [16]. The counts of cell populations and the levels of serum or plasmatic inflammatory proteins were not significantly associated with the SNPs of interest.

In conclusion, we identified a new genetic association for MM, supported by functional biological explanations, thus highlighting the importance of secondary analysis using functional approaches for GWAS.

### Supplementary information

Supplementary methods

Supplementary table 2

## Data Availability

The primary data for this work will be made available to researchers who submit a reasonable request to the corresponding author, conditional to approval by the Steering Committees of the respective studies (InterLymph, GMMG, IMMEnSE) and Ethics Commission of the Medical Faculty of the University of Heidelberg, Germany. Data will be stripped from all information allowing identification of study participants. The summary statistics of the FinnGen GWAS are publicly available at https://www.finngen.fi. Functional data of the Human Functional Genomics Project used in this project have been catalogued and archived in the BBMRI-NL data infrastructure (https://hfgp.bbmri.nl/) using the MOLGENIS open-source platform for scientific data, which allows flexible data querying and download, including sufficiently rich metadata and interfaces for machine processing (R statistics, REST API) and using FAIR principles to optimize Findability, Accessibility, Interoperability and Reusability.
