# Supplementary material for: Identification of novel genetic loci for risk of multiple myeloma by functional annotation
Source: Leukemia. 2023 Sep 18;37(11):2326–9. doi: 10.1038/s41375-023-02022-8 (PMC10624610; doi:10.1038/s41375-023-02022-8)

**Supplementary Material:**

**Supplementary Methods**

**Supplementary table 1**

**Supplementary table 2**

**Supplementary table 3**

**Supplementary figure 1**

**Study populations**

**Interlymph** (https://epi.grants.cancer.gov/InterLymph/)(1) consists of a MM GWAS of 9 participating studies conducted on individuals of European ancestry (2 434 MM cases and 3 446 controls)(2). Genotyping was performed with multiple platforms (Affymetrix, Human660W-quad Beadchip, and Illumina arrays 610 Quad, Omni5, OmniExpress Beadchip, and OncoArray) and imputed with the Haplotype Reference Consortium. Contributing studies were approved by local ethics review committees, and all participants provided written informed consent. Further details of this GWAS study are provided elsewhere^2^.

**GMMG GWAS.** The GMMG GWAS comprises 1 512 MM cases coordinated by the University Clinic of Heidelberg and 2 107 controls sampled from healthy individuals enrolled in the Heinz Nixdorf Recall (HNR) study(3). Genotyping of cases was performed with Illumina Human OmniExpress12 v1.0 arrays, while either Illumina HumanOmni1-Quad_v1 or OmniExpress-12 v1.0 arrays were used to genotype the controls. Genotyping data were imputed using 1000 Genomes Project data. Collection of patient samples and associated clinicopathological information was undertaken with written informed consent and relevant ethical review board approval from the Ethics Commission of the Medical Faculty of the University of Heidelberg, in accordance with the tenets of the Declaration of Helsinki. Diagnosis of MM in all cases was established in accordance with World Health Organization (WHO) guidelines.

**IMMEnSE.** The IMMEnSE consortium consists of a case-control study that collected retrospectively samples and data in order to investigate the role of polymorphic variants in MM risk(4). MM cases (1 343) and controls (1 787) from 8 European countries were included in this study. The cases were defined by a confirmed new diagnosis of MM between 1992 and 2017, using the IMWG diagnostic criteria(4). A retrospective collection of epidemiological and clinico-pathological characteristics, including age, sex, country of origin, disease stage (Durie-Salmon (DS) and/or International Staging System (ISS)), was obtained from medical records of the participating institutions. Controls were collected from each participant center among the general population in Italy, blood donors from Denmark, France, Poland, Portugal, and Spain, and hospitalized subjects with varying diagnoses excluding cancer from Hungary and Spain. The control samples were mostly collected in the same geographic areas as the cases. Age distribution and sex ratio were similar between cases and controls, without individual matching. Written informed consent was obtained from each participant, with approval for collection and use of samples from local Institutional Review Boards and the Ethics Commission of the Medical Faculty of the University of Heidelberg.

**Finngen.** The FinnGen project(5) is a cohort of 309 154 Finnish individuals genotyped with Illumina and Affymetrix platforms (https://www.finngen.fi). Subsequently, imputation was conducted using the SISu v3 reference panel with Beagle 4.1 (version 08Jun17.d8b). The associations of 16 962 023 SNPs with 3 095 endpoints (including MM risk) were tested. Analyses were adjusted for age, sex, 10 principal components (PCs) and for genotyping batch. A total of 693 MM cases and 259 712 controls who were cancer-free at recruitment were evaluated in this study. Summary statistics of genome-wide associations between SNPs and MM risk were obtained from https://r6.finngen.fi on 01/07/2022.

Features of the study populations are summarized in **Table 1**.

**SNP selection**

**Supplementary figure 1** shows the flowchart of the project. Candidate SNPs to be replicated were selected based on their association with MM risk and their functional role. First, we obtained summary results including odds ratios (OR), 95% confidence intervals (95%-CI), and p-value of the top SNPs of the InterLymph GWAS. Subsequently, all SNPs in the MM data set from InterLymph with p<5x10^-4^ were looked up in the first replication dataset, the GMMG GWAS. We did not consider SNPs that were reported to be associated at genome-wide significance level in previous GWASs. All SNPs with significant p-values (p<0.05) in the GMMG GWAS and ORs going in the same direction in both datasets were selected.

The next step was annotating the selected SNPs for their predicted function, using several suitable bioinformatic tools and databases. First, expression and splicing quantitative trait loci (eQTLs and sQTLs) were searched using the Genotype-Tissue Expression (GTEx) portal(6) in the tissues that are the most relevant for MM risk, namely whole blood and EBV-transformed B-lymphocyte cell lines. GTEx was accessed on 21/03/2021.

Next, the selection of SNPs located in transcription factor binding sites (TFBS) was performed using the SNP2TFBS database (https://ccg.epfl.ch/snp2tfbs/)(7). The SNP2TFBS database contains annotations for SNPs predicted to abolish, create, or modify the affinity of binding to one or more of 200 transcription factors (TFs). The effects of SNPs in the whole genome on TF binding were estimated using position weight matrices (PWM), modeling the binding specificity of the corresponding TF. For each SNP-TF binding, a score was computed in the database, based on the difference between the PWM match scores of the two alleles of the SNP. We downloaded all predicted SNP-TFBS interactions from the SNP2TFBS database (N=2 281 137, involving 1 900 881 unique SNPs) on 21/06/2021. PWM scores in the SNP2TFBS database are specific to each TF and not directly comparable across TFs, therefore for each SNP-TFBS interaction we computed the score percentile within each TF-specific dataset from the SNP2TFBS database. Then we ranked the lists of noteworthy SNP-TFBS interactions according to score percentile.

Next, a list of all 11 857 human long non-coding RNA (lncSNPs) was obtained through the publicly available database NONCODE (http://www.noncode.org), consulted on 22/03/2019. The database uses a Coding Noncoding Index algorithm to discriminate between ncRNA and protein-coding RNA through the coding potential of each transcript(8). To identify all the SNPs included in each of the lncRNA sequences identified through NONCODE, LncRNASNP2 (http://bioinfo.life.hust.edu.cn/lncRNASNP#!/), was used. A final list of 10 205 295 lncSNPs was obtained.

Finally, SNPs that are located within enhancer sites and therefore may affect their function were also considered, using a publicly available resource described by Nasser and colleagues(9) that consists of a genome-wide map of more than six million enhancer–gene connections.

We also considered polymorphisms located in gene coding regions that affect the biochemical properties of proteins (missense, stop-gain and stop-loss SNPs) or alter the codon usage (synonymous SNPs). We obtained lists of coding region SNPs from the NCBI Single Nucleotide Polymorphism (dbSNP) public database.

SNPs affecting miRNA binding sites or within miRNAs seed sequences were excluded from this study since we already investigated them in relation to MM risk(10,11).

The resulting list from all annotations was pruned for linkage disequilibrium (LD) using the LDlink portal(12) (https://ldlink.nci.nih.gov/). Only SNPs with r^2^<0.6 among them were kept. Replication in IMMEnSE and FinnGen was attempted for SNPs showing association with risk in the meta-analysis between InterLymph and GMMG GWAS and at least one *in silico* functional annotation.

The InterLymph GWAS resulted in 4 396 SNPs that showed association with MM risk (p<5x10^-4^). After comparing this SNP list to the GMMG GWAS results and excluding 15 loci known to be associated with MM risk, a total of 136 SNPs fulfilled the criteria (exhibiting p<0.05 in the GMMG GWAS and ORs going in the same direction in both datasets) and were chosen for functional annotation. **Supplementary table 2** shows the details of the 136 SNPs and their predicted functional characterization.

We grouped the 136 selected SNPs by LD (r^2^<0.6), resulting in a total of 14 independent loci on 9 chromosomes. After exclusion of SNPs that had already been analysed in IMMEnSE in the context of previous projects and already shown not to be significantly associated with MM risk (on chromosomes 6, 8, 12 and 21), 4 SNPs showed to have low p-value of association with MM risk and had at least one functional prediction annotation (rs12038685, rs2664188, rs12652920, rs28199) which were therefore chosen for replication in IMMEnSE **(Supplementary table 3).**

**Genotyping and quality control**

Genotyping of MM cases and controls from IMMEnSE was performed with TaqMan technology, in 384-well plates containing similar numbers of cases and controls. Approximately 6% of the samples were duplicated to evaluate concordance rate of the genotyping. Additionally, to assess the quality of the genotyping, the study subject call rate was evaluated. Pearson’s chi-square test (χ^2^) was performed to assess if genotype frequencies were in Hardy-Weinberg equilibrium (HWE). The analysis, restricted to controls, was performed overall and separately for each country.

### Details of genotyping and quality control of GMMG, InterLymph and FinnG en study can be found in the supplementary material.

Data of the GMMG and InterLymph GWAS were separately imputed with the IMPUTE2 v2.3 software or the Michigan imputation server (using the Haplotype Reference Consortium data), respectively(13). Post-imputation quality control measures included filtering for high-quality imputation results (INFO score >0.8), missingness rate, checks for duplicates, abnormal heterozygosity, cryptic relatedness, population outliers (evaluated by principal components analyses using Eigenstrat software), and genomic inflation (evaluated by the λ parameter).

Genotyping, imputation and quality control for FinnGen were previously described in detail(5). Briefly, samples of FinnGen participants were genotyped with SNP arrays by Illumina and Affymetrix. Standard quality control was performed checking genetic sex, genotype missingness, heterozygosity, and HWE. Imputation was performed with Beagle 4.1 using a reference panel specific for the Finnish population. After imputation, variants with INFO score < 0.6 or MAF < 0.0001 were excluded.

1. Morton LM, Sampson JN, Cerhan JR, Turner JJ, Vajdic CM, Wang SS, et al. Rationale and Design of the International Lymphoma Epidemiology Consortium (InterLymph) Non-Hodgkin Lymphoma Subtypes Project. J Natl Cancer Inst Monographs. 2014 Aug;2014(48):1–14.

2. Clay-Gilmour AI, Hildebrandt MAT, Brown EE, Hofmann JN, Spinelli JJ, Giles GG, et al. Coinherited genetics of multiple myeloma and its precursor, monoclonal gammopathy of undetermined significance. Blood Adv. 2020 Jun 23;4(12):2789–97.

3. Mitchell JS, Li N, Weinhold N, Försti A, Ali M, van Duin M, et al. Genome-wide association study identifies multiple susceptibility loci for multiple myeloma. Nat Commun. 2016 01;7:12050.

4. Martino A, Sainz J, Buda G, Jamroziak K, Reis RM, García-Sanz R, et al. Genetics and molecular epidemiology of multiple myeloma: the rationale for the IMMEnSE consortium (review). Int J Oncol. 2012 Mar;40(3):625–38.

5. Kurki MI, Karjalainen J, Palta P, Sipilä TP, Kristiansson K, Donner K, et al. FinnGen: Unique genetic insights from combining isolated population and national health register data [Internet]. medRxiv; 2022 [cited 2022 Jul 19]. p. 2022.03.03.22271360. Available from: https://www.medrxiv.org/content/10.1101/2022.03.03.22271360v1

6. Consortium TGte. The GTEx Consortium atlas of genetic regulatory effects across human tissues. Science. 2020 Sep 11;369(6509):1318–30.

7. Kumar S, Ambrosini G, Bucher P. SNP2TFBS - a database of regulatory SNPs affecting predicted transcription factor binding site affinity. Nucleic Acids Res. 2017 Jan 4;45(D1):D139–44.

8. Zhao Y, Li H, Fang S, Kang Y, wu W, Hao Y, et al. NONCODE 2016: an informative and valuable data source of long non-coding RNAs. Nucleic Acids Res. 2016 Jan 4;44(Database issue):D203–8.

9. Nasser J, Bergman DT, Fulco CP, Guckelberger P, Doughty BR, Patwardhan TA, et al. Genome-wide enhancer maps link risk variants to disease genes. Nature. 2021 May 13;593(7858):238–43.

10. Macauda A, Calvetti D, Maccari G, Hemminki K, Försti A, Goldschmidt H, et al. Identification of miRSNPs associated with the risk of multiple myeloma. International Journal of Cancer. 2017;140(3).

11. Melaiu O, Macauda A, Sainz J, Calvetti D, Facioni MS, Maccari G, et al. Common gene variants within 3’-untranslated regions as modulators of multiple myeloma risk and survival. Int J Cancer. 2021 Apr 15;148(8):1887–94.

12. Machiela MJ, Chanock SJ. LDlink: a web-based application for exploring population-specific haplotype structure and linking correlated alleles of possible functional variants. Bioinformatics. 2015 Nov 1;31(21):3555–7.

13. Howie BN, Donnelly P, Marchini J. A flexible and accurate genotype imputation method for the next generation of genome-wide association studies. PLoS Genet. 2009 Jun;5(6):e1000529.

**Tables and Figures**

**Supplementary table 1.** Study populations.

|  | **InterLymph** | **GMMG** | **FinnGen^a^** | **IMMEnSE** | **Total** |
| --- | --- | --- | --- | --- | --- |
| **Number of subjects** |  |  |  |  |  |
| Cases | 2 434 | 1 512 | 693 | 1 343 | 5 982 |
| Controls | 2 567 | 2 107 | 259 712 | 1 787 | 266 173 |
| **Median age** | 62 (54-69) | 59^b^ | - | 62 (55-69) |  |
| **Sex** |  |  |  |  |  |
| Males | 58% | 57%^c^ | - | 51% |  |
| Females | 42% | 43%^c^ | - | 49% |  |

^a^ Details on age and sex distribution of FinnGen individuals are not available.

^b A^vailable only for MM cases.

**Supplementary table 3.** Selected SNPs for replication^a^.

| **rsID** | **Location^b^** | **OR^c^** | **95%C.I.^d^** | **p-value**  **meta-analysis** | **p-value het^e^** | **Functional annotation^f^** |
| --- | --- | --- | --- | --- | --- | --- |
| rs12038685 | Chr 1: 165 216 540 | 1.17 | 1.08-1.25 | 1.56x10^-5^ | 0.192 | TBFS |
| rs2664188 | Chr 4: 40 079 994 | 1.14 | 1.07-1.21 | 2.31x10^-5^ | 0.218 | eQTL |
| rs12652920 | Chr 5: 132 549 548 | 0.81 | 0.80-0.92 | 2.65x10^-5^ | 0.275 | TBFS |
| rs28199 | Chr 5: 173 150 766 | 1.16 | 1.10-1.23 | 9.97x10^-7^ | 0.587 | TBFS |

^a^ Results are referred to the meta-analysis between InterLymph and GMMG GWAS datasets

^b^ Location according to hg38

^c^ OR=odds ratio

^d^ 95%C.I.= 95% confidence interval

^e^ p-value het= p value of the heterogeneity test between study

^f^ TFBS: the SNP is predicted to alter one or more transcription factor binding site(s); eQTL: the SNP is predicted to be a quantitative trait locus in whole blood or EBV-transformed B-lymphocyte cell lines

**Supplementary figure 1.** Workflow of the study.


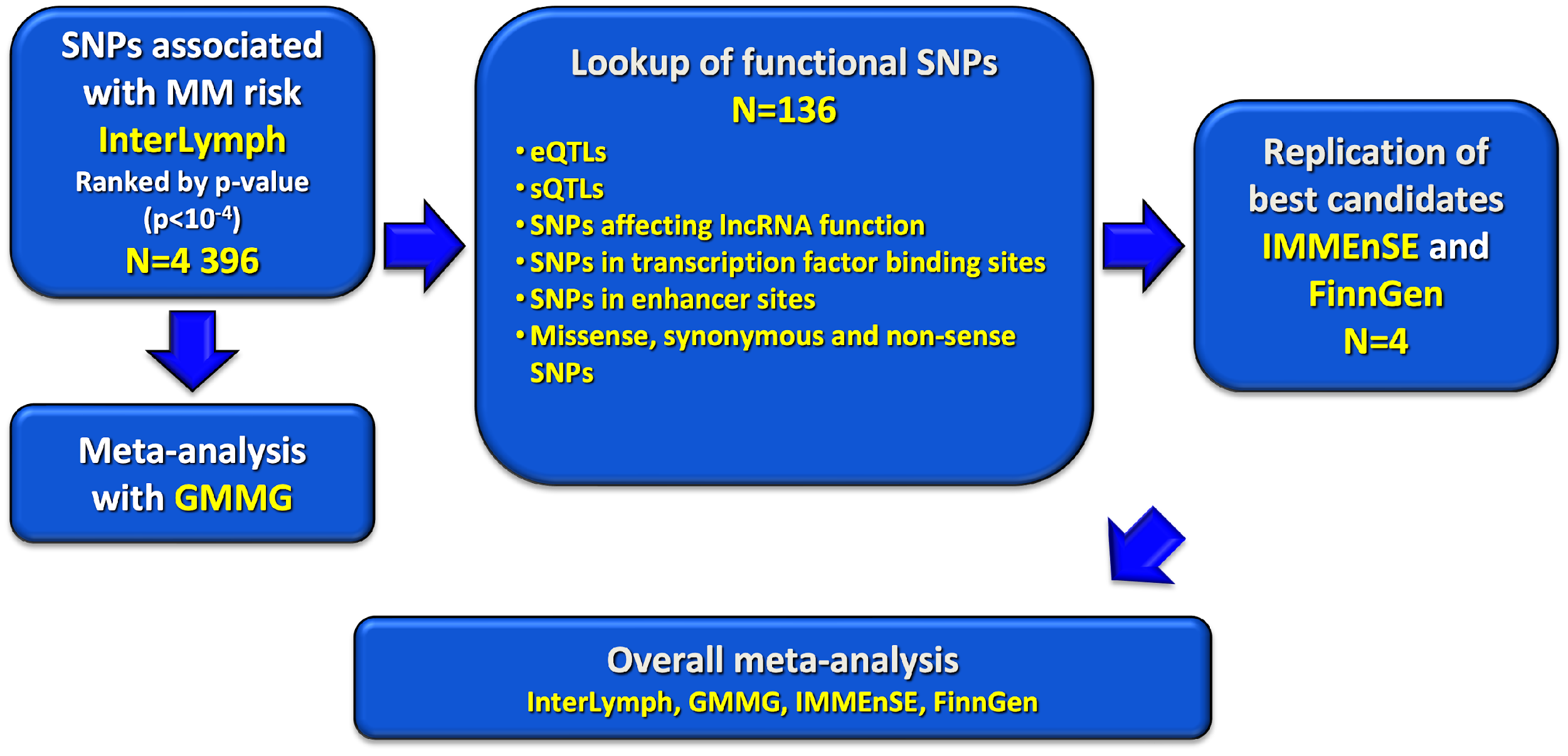

Supplement: Supplementary file 1 — Supplementary methods [file 41375_2023_2022_MOESM1_ESM.docx]
